# Numerical simulation of tropical cyclone generated waves in South China Sea during winter monsoon surge

**DOI:** 10.1038/s41598-020-79204-2

**Published:** 2020-12-17

**Authors:** Peng Qi, Aimei Wang

**Affiliations:** 1 CAS Key Laboratory of Ocean Circulation and Waves, Institute of Oceanology, Chinese Academy of Sciences (CAS), Qingdao, 266071 China; 2 Center for Ocean Mega-Science, Chinese Academy of Sciences, Qingdao, 266071 China; 3 Laboratory for Ocean and Climate Dynamics, Qingdao National Laboratory for Marine Science and Technology, Qingdao, 266237 China; 4 National Marine Information Center, Tianjin, 300171 China

**Keywords:** Natural hazards, Physical oceanography

## Abstract

The South China Sea (SCS) is a highly semi-enclosed marginal sea located in the East Asian monsoon region. This paper proposes interesting aspects of the unique feature of the SCS waves in response to tropical cyclone's passage when large-scale winter monsoon winds prevail. We use the wave model WaveWatch III to study the wave characteristics of typhoon Durian (2006) passing over the middle of SCS in early December 2006, and state the new understanding acquired in the aspects of the tropical cyclone generated waves in the SCS during winter monsoon surge. In light of this, the role of the large-scale NE monsoon winds on winter typhoon wave field characteristics in the SCS are highlighted by conducting sensitivity experiments with and without the NE monsoon winds. The NE monsoon winds weakly affect the SWH field near the typhoon track and strongly away from the track, especially in the deep water area of the northern SCS where the NE monsoon winds produce high waves. Comparisons between the two experiments show the effect of the NE monsoon winds on the directional wave spectra in the SCS, suggesting that the monsoon-generated swells do not decay and remain throughout the typhoon period.

## Introduction

The South China Sea (SCS) is located at 0°–23° N, 99° E–121° E, with a total area of about 3.5 × 10^6^ km^2^. It is the largest and highly semi-enclosed marginal sea between the Asian continent to the north and the west, the Philippine islands to the east, and the Kalimantan (Borneo) to the south. Its central basin is similar to an elliptical shape: the major axis (southwest–northeast) is about 1900 km, and the minor about 1100 km. The central depth is above 4000 m. The SCS is connected to the surrounding oceans by several straits: the western Pacific through Luzon Strait, the southern shelf of the East China Sea through Taiwan Strait, the Java Sea through Gasper and Karimata Straits, and to the Indian Ocean through the Straits of Malacca. All of these straits are shallow except the Luzon Strait, the maximum depth of which is around 2000 m^1^. As the “Maritime Silk Road” of the “Belt and Road” today, the SCS has been the most important waterway to the Indian Ocean between China and foreign trade and culture since ancient times. The SCS is also one of the major tropical fisheries and quality fisheries in China^2^.

The uniqueness of the SCS lies not only in its semi-closure, but in the property of both Asian monsoon and typhoons. Northeasterly (NE) winds prevail from November to March for winter monsoon, southwesterly (SW) from April to August for summer monsoon. Winter monsoon winds are significantly stronger than summer. The SCS is also attacked by tropical cyclone-TC (typhoon or hurricane in the U.S.) mainly from May to December. TC passing by the SCS can be divided into two categories by the source area where they initiate: the western Pacific TC generated on the vast tropical western Pacific Ocean surface east of the Philippines and the SCS native TC mainly generated in the central and eastern SCS. The average annual tropical cyclone frequency in the SCS is about 10 according to the “Tropical Cyclones Best Track Data (1959–2014)” provided by the “China Meteorological Administration”. Besides, a TC can pass by the SCS in all the months within a year^3^. The primary reason is the persistently warm sea surface temperature and the location of the intertropical convergence zone (ITCZ). About 80% of the TCs are initially generated in the monsoon trough^3^.

A TC with violent and rapidly changing winds generates heavy and complicated wave fields near the vortex center, resulting in dramatic variations of the wave field in a larger spatial extent^4^. The ERS-1 satellite imagery from synthetic aperture radar (SAR) for tracking the wave field induced by an intense storm was examined and compared with both buoy data and model results^5^. The effect of fetch on the directional spectrum of Celtic Sea storm waves was investigated using high-frequency radar measurements of directional spectra^6^. Variations of the directional wave spectrum of Hurricane Bonnie (1998) in the Atlantic Ocean were studied for both open ocean^7^ and landfall cases^8^ using the NASA Scanning Radar Altimeter (SRA) for the first time when it passed in the western Atlantic Ocean. A numerical simulation experiment for sea surface directional wave spectra under the wind forcing of Hurricane Bonnie (1998) was carried out by running the third-generation wave model WAVEWATCH III (hereafter referred to as NWW3), and validated by buoy observations and SRA data^9^. It is shown that the hurricane-generated waves are determined by the distance from the hurricane center or radius of maximum winds (*R*_max_) as well as the hurricane moving velocity. However, the western Atlantic Ocean is a non-monsoon. The SCS, which is a highly semi-enclosed marginal sea, is located in the world-renowned East Asian monsoon area. Strong waves occur frequently in winter in the SCS. Therefore, the effects of the typhoon and monsoon combined wind forcing on the wave characteristics are unique in the SCS, and may not be representative of the western North Atlantic^10^.

Observation buoys provide reliable observations of ocean surface winds, especially as a means of TC maximum wind speed acquisition. However, available buoys are quite limited and isolated at present. On the other hand, a TC surface wind field can be produced by the mesoscale atmospheric numerical prediction model with many approximations in the air-sea interfaces. It is, however, known that TC surface winds produced by the mesoscale atmospheric model are not very accurate because people have not yet fully grasped all their intricacies and implications^11−13^. Recent advances in remote sensing are powerful in acquisition of surface winds over the global oceans. The Cross-Calibrated Multi-Platform (CCMP) remotely sensed wind produced and distributed by AVISO is a newly released ocean wind dataset covering the global ocean surface and of high accuracy. The CCMP, after all, is large-scale wind. It may underestimate the maximum velocity near the TC center^14^. In practical applications, engineers and scientists often resort to the use of a parametric model to approximate the surface wind structure of TC. A TC wind field is generally confined within 100 km of the vortex center. The wind is calm at the center of the eye. It increases rapidly with radius, reaching a maximum at the outer edge of the cloud-free eye. Outside the eye, the wind decreases with radius, not always monotonically, and approaches zero at several hundred to 1000 km from the center based on an idealized single TC model without considering the background wind field. A parametric TC model reflecting radial variation of the hurricane pressure was proposed^15^ to compute the gradient-balance wind. Later, a pressure-wind relation was derived^16^, in which the maximum balanced wind is proportional to the square root of the pressure difference between the radius peripheral and the center. The Schloemer model was used in the wind input to the Sea, Lake and Overland Surges from Hurricanes (SLOSH) storm-surge model^17−19^ and to the modeling of wind-driven waves and other oceanic responses to hurricanes (e.g., literature^20^). There are two key parameters in both the Schloemer model and the Jalesnianski model^17,18^, the maximum wind, *V*_max_, and the radius of the maximum wind, *R*_max_. A third parameter *B* was added^21^, which controls the radial width of the wind maximum and is helpful in the inner core region. However, the Holland model can not accurately give the outer TC wind field due to its extension of the vortex wind field to infinity^22,23^.

In this article, we carried out a case study of typhoon (TY) Durian (2006) when it passed over the middle of the SCS in early December 2006. First of all, the importance of this work lies in that the SCS is both a highly semi-enclosed marginal sea and part of the world-renowned east Asian monsoon climate zone. Here over the SCS, not only winter monsoon stronger and fast moving southeasterly in conjunction with cold air outbreaks (cold surge), but also late autumn and winter typhoons passing the middle of the SCS more frequent. Nature has provided us with the unique natural experimental fields in the SCS for the typhoon wave overlapping monsoon swell research. Our goal is to verify the unique feature of monsoon, typhoon, and monsoon again wave fields. The outline of the paper is as follows. “Typhoon winds” section delineates the parametric typhoon wind model of Jalesnianski and the processing technique for a smooth transition from typhoon winds to environmental winds. “Numerical experiments of typhoon generated waves with NE monsoon winds” section depicts model settings for typhoon waves in conjunction with NE monsoon wind forcing. “Results and discussions” section describes wave characteristics including SWH and directional wave spectra, and discusses the effects of monsoon winds on the typhoon wave characteristics. “Conclusions” section gives the conclusions.

## Typhoon winds

### A parametric typhoon wind model

For a traveling typhoon, the moving velocity of the typhoon center should be added to the vortex wind velocity. Let ***V***_c_ be the wind vector relative to the typhoon center and ***V***_m_ be the moving velocity. Consider an idealized circular storm, symmetric in wind velocity about its center, with maximum wind velocity $V_{\max }$ at distance $R_{\max }$ from its center, and consider the moving velocity of the storm center with components $\left( {V_{{{\text{ox}}}} , \, V_{{{\text{oy}}}} } \right)$, the parametric wind field model of the moving storm is defined as^17,18,23^,

1 $$  \varvec{V} = \varvec{V}_{{\text{c}}}  + \varvec{V}_{{\text{m}}}  = \left\{ {\begin{array}{*{20}l}    {V_{{{\text{max}}}} \frac{{2r/R_{{\max }} }}{{1 + \left( {r/R_{{\max }} } \right)^{2} }}\frac{1}{r}\left( {A\varvec{i} + B\varvec{j}} \right) + ~\frac{r}{{R_{{{\text{max}}}}  + r}}\left( {V_{{{\text{ox}}}} \varvec{i} + V_{{{\text{oy}}}} \varvec{j}} \right){{,}}} \hfill & {r \le R_{{{\text{max}}}} } \hfill  \\    {V_{{{\text{max}}}} \frac{{2r/R_{{\max }} }}{{1 + \left( {r/R_{{\max }} } \right)^{2} }}\frac{1}{r}\left( {A\varvec{i} + B\varvec{j}} \right) + \frac{{R_{{{\text{max}}}} }}{{R_{{{\text{max}}}}  + r}}\left( {V_{{{\text{ox}}}} \varvec{i} + V_{{{\text{oy}}}} \varvec{j}} \right)~{{,}}} \hfill & {r > R_{{{\text{max}}}} } \hfill  \\   \end{array} } \right.  $$

where ${\varvec{V}}$ on the left side is the wind velocity at a distance $r$ from the center of the storm. $\left( {{\varvec{i}},{\varvec{j}}} \right)$ are unit vectors on the coordinate axis $\left( {x,y} \right)$, respectively. Parameters $A$ and $B$ are expressed, respectively, $A = - \left( {y - y_{{\text{c}}} } \right)\cos \theta - \left( {x - x_{{\text{c}}} } \right)\sin \theta$, $B = \left( {x - x_{{\text{c}}} } \right)\cos \theta - \left( {y - y_{{\text{c}}} } \right)\sin \theta$, where $\left( {x_{{\text{c}}} , \, y_{{\text{c}}} } \right)$ denote locations of the storm center. $\theta$ is the ingress angle. In the surface wind field of a typhoon, the wind in general is directed across the isobars into the interior of the cyclone due to friction on a coarse sea surface. This ingress angle varies in space and time. According to previous studies^24,25^ and for simplicity, $\theta$ is taken as constant and *θ* = 20°.

The axisymmetric wind fields generated by the parametric TC wind model correspond to boundary layer averaged winds above the surface. The computed winds are adjusted to the 10 m level (${\varvec{V}}_{{{10}}}$) using

2 $$ \left| {{\varvec{V}}_{10} } \right| = K_{{\text{m}}} \left| {{\varvec{V}}_{{}} } \right| $$

where $K_{{\text{m}}}$ is a correction factor. Based on GPS dropwindsonde measurements, $K_{{\text{m}}} = 0.8$ is used as in the SLOSH and Rankine models^25^.

The radius of maximum wind $R_{\max }$ is a key parameter that determines the spatial scale of a typhoon vortex wind field in Eq. (1). Willoughby and Rahn (2004)^26^ found that the frequency distribution of $R_{\max }$ exhibits a substantial tail on the large-radius side of the mean, and proposed an expression of the radius of maximum wind as a function of the maximum wind and latitude $\phi$. Scaling of $R_{\max }$ by Eq. (3) narrows the distribution, reducing the logarithmic standard deviation^26^.

3 $$ R_{{{\text{max}}}} = 51.6\exp \left( { - 0.0223V_{{{\text{max}}}} + 0.0281\phi } \right) $$

### Transition from the typhoon vortex to the background winds

**Figure 1:**
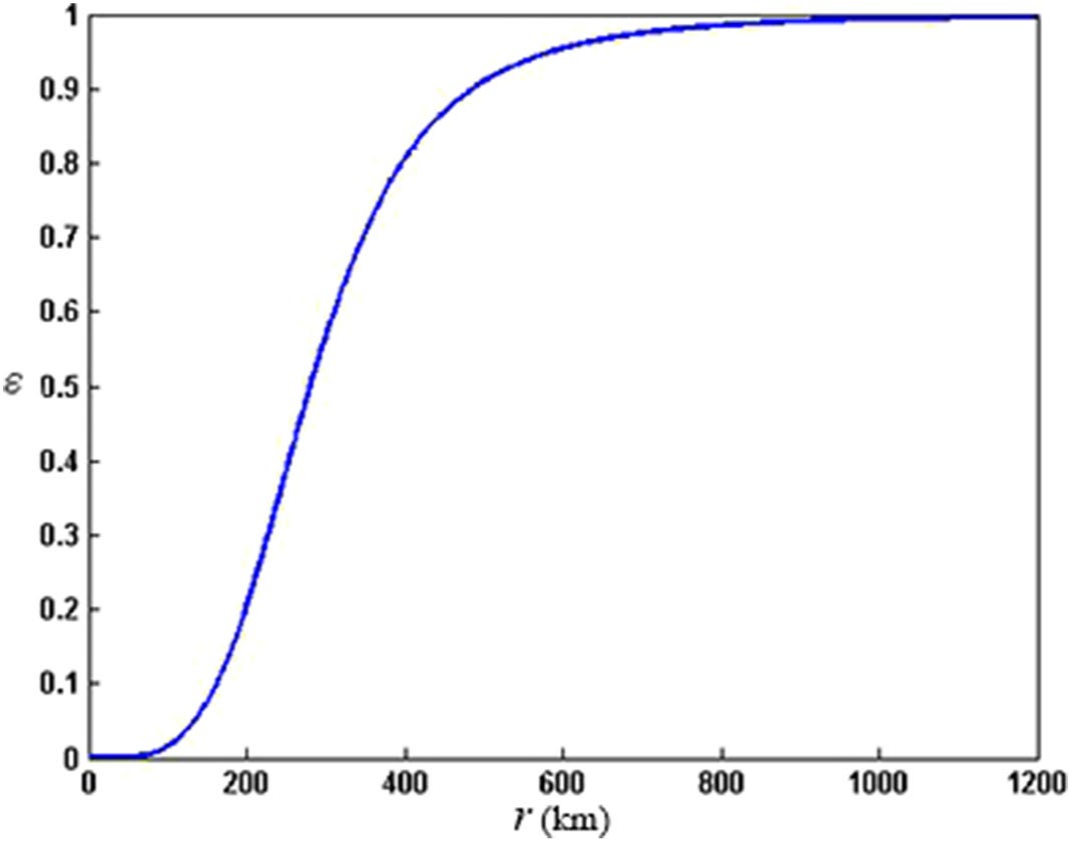
$$\varepsilon$$ varies with $$r$$.

The parametric model wind field of a moving storm is added to large-scale background winds of monsoon. To avoid discontinuousness between the peripheral winds of the typhoon vortex system and the background winds of the monsoon, the following weighted average algorithm is used for the sake of smoothing,

4 $$ {\varvec{V}}_{{{\text{bl}}}} = \left( {1 - \varepsilon } \right){\varvec{V}}_{10} + \varepsilon {\varvec{V}}_{{{\text{bg}}}} $$

where ${\varvec{V}}_{{{\text{bg}}}}$ and ${\varvec{V}}_{{{\text{bl}}}}$ denote the background and the blended winds, respectively. $\varepsilon$ is the weight,

5 $$ \varepsilon = \frac{{c^{4} }}{{1 + c^{4} }},\quad c = \frac{r}{{nR_{\max } }} $$

where *c* is an intermediate variable, *r* denotes the radial distance from the typhoon center (unit: km). Here $n = 9$. $\varepsilon$ varies with $r$ is shown in Fig. 1.

## Numerical experiments of typhoon generated waves with NE monsoon winds

### Wave model settings

The third generation wave model NWW3 is used in this paper. We take the SCS as our target computational domain (i.e., 0°–25° N, 105° E–122° E). Bathymetry data from ETOPO5 database (provided by the U.S. National Geophysical Data Center) are utilized. The mesh is generated with 0.25° interval horizontal grids, 25 frequency intervals in the wave number grid as (10), and 24 directional bands spaced at 15° with the first band centered at 0°.

10 $$ \sigma_{m + 1} = 1.1\sigma_{m \, } , \, m = 0,1,2, \ldots ,24 $$

with the first frequency as 0.0418 Hz.

The marching-on-in-time algorithm (MOT) in the NWW3 model integration comprises the following four time steps, that is, global time step (300), spatial time step (300), spectral time step (300), and source time step (100). Shown in parenthesis are the time step (unit: seconds) we used in the MOT for the sake of accuracy and efficiency.

In viewing of TY Durian genesis east of the Philippines on November 26, entering the SCS on December 1, and moving out of the SCS on December 5, we began the integration of the NWW3 for typhoon-monsoon waves in the SCS at 0000UTC November 25 (5 days earlier than the typhoon’s entry time, see “Typhoon Durian (2006)” section) and ended it to at 0000UTC December 6.

### Typhoon Durian (2006)

**Figure 2:**
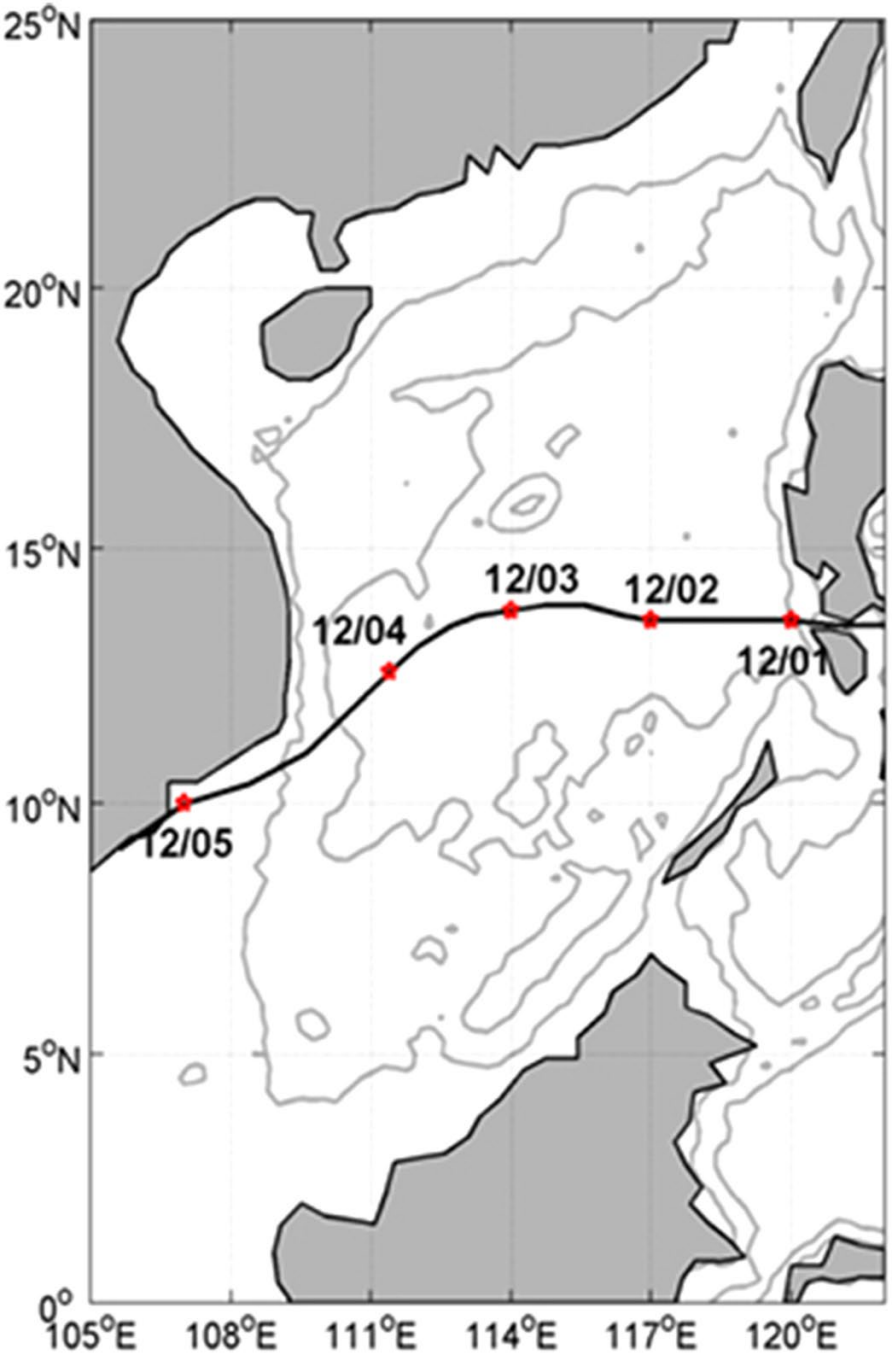
TY Durian's track in the SCS (Red dots denote the location of the typhoon center at 0000 UTC).

Typhoon Durian (2006) was first developed as a tropical depression (TD) on November 25, 2006 over the tropical western Pacific Ocean (9.6° N, 146.1° E). Guided by large-scale easterly winds, it moved westward across the tropical Pacific Ocean to the Philippines and was steadily upgraded (Table 1). From tropical storm (TS) to Super TY, Durian showed rapid intensification in east of the Philippines before November 30. On November 30, Durian made landfall and traversed central Luzon Island to the west as it weakened a little. It entered the SCS and was weakened to 40 m/s at 0000UTC on December 1 due to the surface friction. Its intensity was further decreased to 35 m/s, still TY grade. Affected by large-scale NE monsoon winds over the whole SCS, from December 3, Durian slowly headed towards the southwest (Fig. 2). From December 4, it downgraded to a severe tropical storm (STS) and was approaching to the southern part of Vietnam. Durian winds quickly dropped to 15 m/s at 0600UTC on December 5, downgraded to TD about 90 km south of Ho-Chi-Minh City in the south of Vietnam, and made landfall at a place in Mekong Delta, and then the TD entered and died in the middle of the Gulf of Thailand. The records in Table 1 and values of the parameter $V_{\max }$ and $\left( {V_{{{\text{ox}}}} , \, V_{{{\text{oy}}}} } \right)$ in Eqs. (1) and (3) are taken from the “Tropical Cyclones Best Track Data (1959–2014)” China Meteorological Administration (2015).

**Table 1:** Best track record of TY Durian (2006) from 0600UTC November 25 to 0600UTC December 5 2006.

Date (mn/dt/yr)	Time (UTC)	Lon (°E)	Lat (N)	Type	*V*_m_ (m/s)	*P*_o_ (hPa)	*V*_max_ (m/s)	*R*_max_ (km)
11/25/2006	1800	145.1	9.6	TD	5.10	1000	15	48.37
11/26/2006	0000	144.0	9.9	TD	5.79	1000	15	48.77
11/26/2006	0600	142.8	10.1	TS	6.17	995	18	45.87
11/26/2006	1200	141.6	10.3	TS	6.16	995	20	44.12
11/26/2006	1800	140.5	10.5	TS	5.66	995	20	44.37
11/27/2006	0000	139.7	10.6	TS	4.08	995	20	44.50
11/27/2006	0600	138.7	10.7	TS	5.08	990	23	41.73
11/27/2006	1200	137.7	10.8	TS	5.08	990	23	41.85
11/27/2006	1800	136.5	11.0	STS	6.15	990	25	40.25
11/28/2006	0000	134.7	11.3	STS	9.22	990	25	40.59
11/28/2006	0600	133.1	11.9	STS	8.63	980	30	36.93
11/28/2006	1200	131.6	12.2	TY	7.70	975	33	34.83
11/28/2006	1800	130.1	12.6	TY	7.81	970	35	33.69
11/29/2006	0000	128.6	13.0	TY	7.80	960	40	30.47
11/29/2006	0600	127.3	13.2	SuperTY	6.59	935	55	21.93
11/29/2006	1200	126.4	13.3	SuperTY	4.54	920	60	19.67
11/29/2006	1800	125.4	13.4	SuperTY	5.03	935	55	22.06
11/30/2006	0000	124.6	13.4	SuperTY	4.00	935	55	22.06
11/30/2006	0600	123.6	13.4	STY	5.00	940	50	24.66
11/30/2006	1200	122.4	13.5	STY	6.03	940	50	24.73
11/30/2006	1800	121.2	13.5	STY	6.00	950	45	27.64
12/01/2006	0000	120.0	13.6	TY	6.02	960	40	31.00
12/01/2006	0600	119.2	13.6	TY	4.00	970	35	34.65
12/01/2006	1200	118.6	13.6	TY	3.00	970	35	34.65
12/01/2006	1800	117.7	13.6	TY	4.50	970	35	34.65
12/02/2006	0000	117.0	13.6	TY	3.50	970	35	34.65
12/02/2006	0600	116.4	13.7	TY	3.04	970	35	34.74
12/02/2006	1200	115.6	13.9	TY	4.13	970	35	34.94
12/02/2006	1800	114.7	13.9	TY	4.49	960	40	31.25
12/03/2006	0000	114.0	13.8	TY	3.53	960	40	31.17
12/03/2006	0600	113.3	13.7	TY	3.54	960	40	31.08
12/03/2006	1200	112.7	13.5	TY	3.17	960	40	30.90
12/03/2006	1800	112.0	13.1	TY	4.06	975	33	35.72
12/04/2006	0000	111.4	12.6	STS	3.96	980	30	37.66
12/04/2006	0600	110.5	11.8	STS	6.12	990	25	41.17
12/04/2006	1200	109.6	11.0	STS	6.13	985	28	37.65
12/04/2006	1800	108.4	10.4	STS	6.81	985	28	37.02
12/05/2006	0000	107.0	10.0	TS	7.38	990	23	40.92
12/05/2006	0600	105.6	9.1	TD	8.48	1000	15	47.69

Here *V*_m_ is the moving velocity of the typhoon center, *P*_o_ the central pressure, *V*_max_ the maximum wind speed, and *R*_max_ the radius of *V*_max_.

Environmental surface winds [in Eq. (4)] are from the CCMP with spatial and temporal resolutions of the winds 0.25° × 0.25° and 6-h, respectively. Quality control was implemented before the usage of the data. Along-track winds of altimeter products from Jason-1 and Jason-2 satellites were used for validations^14^. The environmental surface wind fields over the SCS are dominated by NE monsoon winds in early December 2006.

**Figure 3:**
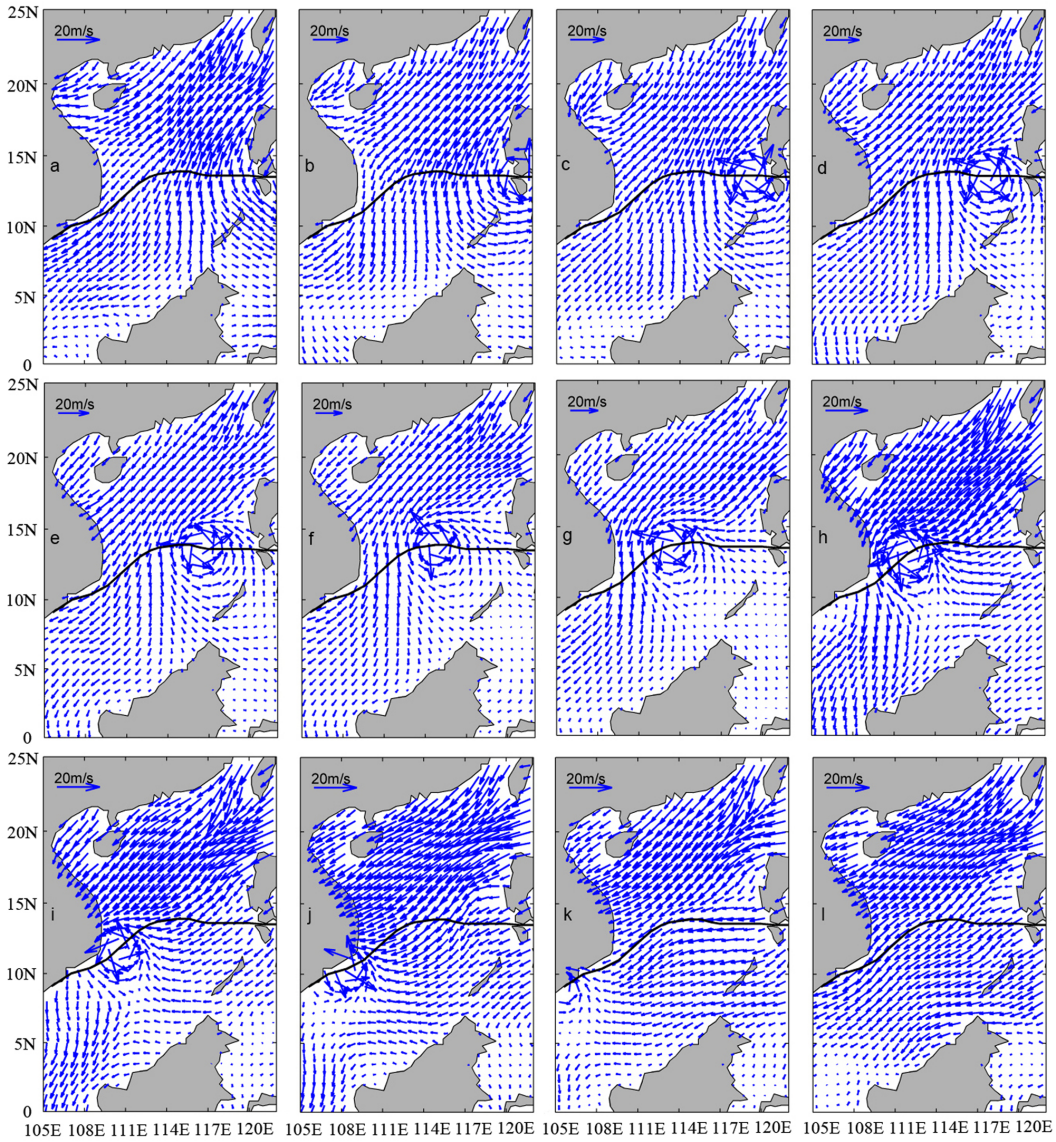
Twelve-hour evolution of the blended wind vector fields from 0600UTC November 30 to 1800UTC December 5.

The blended wind fields over the SCS in early December 2006 during the passage of TY Durian (2006) are established using Eq. (4). A twelve-hour evolution of the blended wind vector fields from 0600UTC November 30 to 1800UTC December 5 is shown in Fig. 3. Here, Fig. 3a in the upper panel shows the wind field before TY Durian entered the SCS, and Fig. 3l in the lower panel the one after the typhoon moved out from the SCS. As we know, the SCS is one of the most active monsoon areas in the world. Winter monsoon winds are mainly from the north air current of Mongolia High. Because of influence of the Coriolis force and position of the SCS to the High, winds of NE and ENE are prevalent over the SCS. Figure 3c –j represent the period during Durian’s passage through the SCS. It is necessary to validate the blended wind fields with independent data. To do so, we used the along-track altimeter wind measurements of Jason-1 satellite in the period of TY Durian’s passage in the SCS. We computed mean bias, root-mean-square (RMS) error and correlation coefficient (CC). Examining the statistics, the mean bias of the two datasets is − 0.12 m/s (namely, the blended winds are underestimated) with a RMS error of 0.79 m/s and CC 0.87. The statistics suggests that the typhoon-monsoon blended wind field during the period of TY Durian is reasonably good. Xie et al.^23^ computed wind fields of northwestern Pacific typhoons Ellen (1983), Sally (1996), Dujuan (2003) and Wipha (2007) using five different parametric tropical cyclone wind models, respectively. By intercomparison and comparison with measured data, the effectiveness and applicability of Jelesnianski wind model were validated.

**Figure 4:**
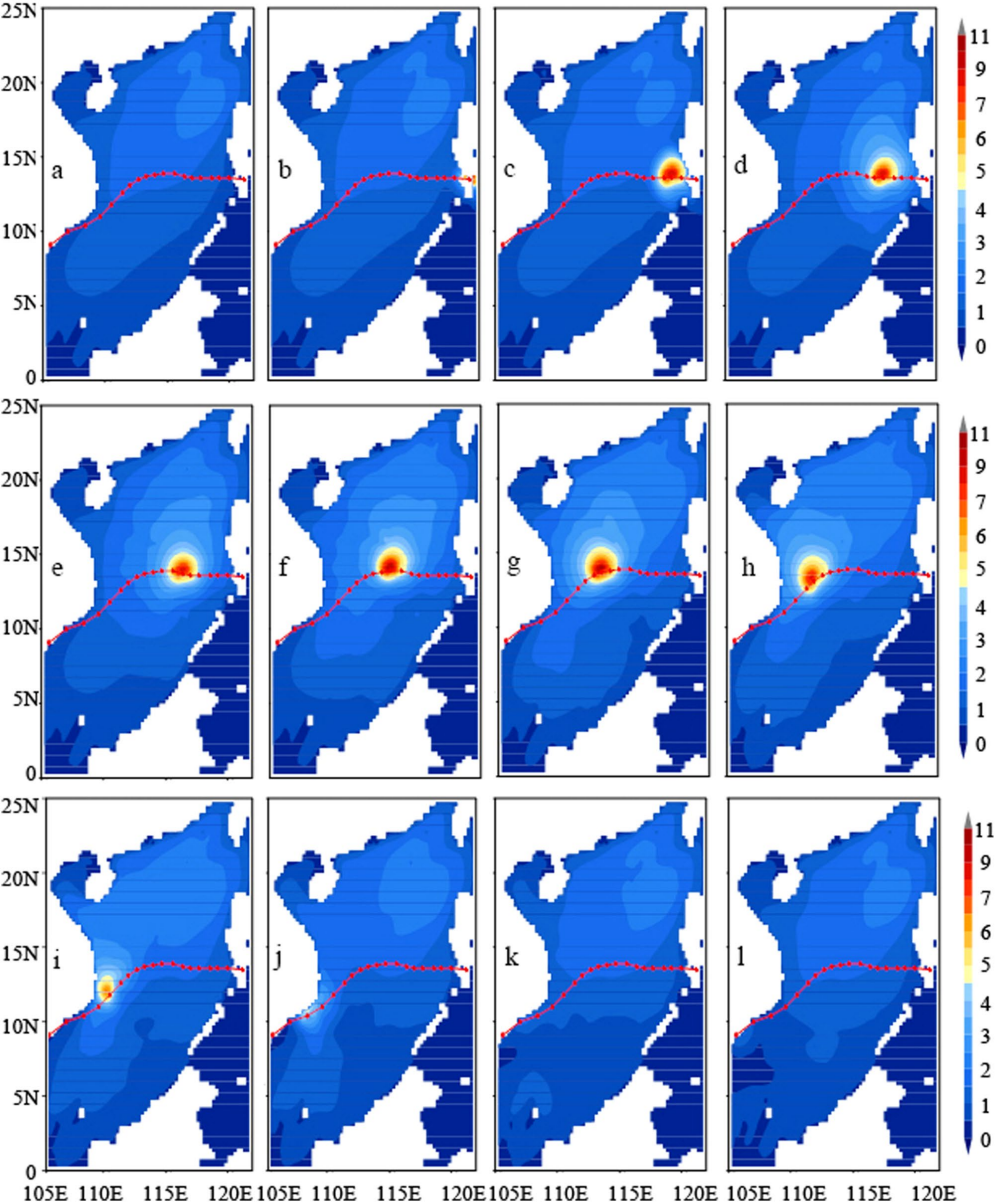
Twelve-hour evolution of SWH in the SCS during the passage of TY Durian (2006).

Figure 4 shows the twelve-hour evolution of SWH fields over the SCS during the passage of TY Durian (2006). Due to lack of direct-measurement platforms, such as vessels or buoys, during typhoon seasons in the SCS, the along-track altimeter SWH data of satellite Jason-1 in the period of TY Durian’s passage in the SCS were used for model validation. Data for validation are the along-track observations from the track 190, the time passing the SCS at 1200UTC on December 2, 2006. We computed mean bias, RMS error and CC. The statistics show that the mean bias is − 0.29 m (namely, the NWW3 modeled SWHs are underestimated) with a RMS error of 0.52 m and CC 0.95, suggesting that the hindcasting results by the NWW3 model under the combined wind forcing of parametric typhoon model winds and background winds of the SCS winter monsoon are reasonably good. This is in agreement with Xie et al.^23^.

## Results and discussions

The wave model NWW3 was forced by blended wind fields of the parametric model typhoon winds and the large-scale NE monsoon winds [i.e., monsoon winds ***V***_en_ ≠ 0 in Eq. (4)] to hindcast the unique wave characteristics during TY Durian’s passage in the SCS in early December 2006. In this numerical experiment, we integrated the NWW3 model from 0000UTC November 25 (namely, 5 days earlier than the typhoon’s entry time) to 0000UTC December 6 with the blended winds. The significant wave heights (SWH) are influenced by the typhoon and the NE monsoon winds.

### SWH fields

First, SWH is used to reflect the SCS wave characteristics under combined wind forcing of typhoon and winter monsoon winds. SWH fields from 0600UTC November 30 to 1800UTC December 6 are shown in Fig. 4 with twelve snapshots. The model output is every 12 h. Before TY Durian arrival, the SCS surface is forced by steady NE monsoon winds. Obviously, the first two snapshots in the upper panels (Fig. 4a,b), from 0600 to 1800UTC November 30, are monsoon- generated wave fields, which is mainly determined by wind speed, duration and fetch, especially a fetch-limited situation forced by the steady winter monsoon winds.

Next, typhoon-generated waves are displayed by the snapshots from 0600UTC December 1 to 1800UTC December 4 when TY Durian passed in the middle of SCS (Fig. 4d–i). The central pressure of TY Durian reduced to the minimum (960 hPa) in the SCS at 0600UTC on December 3 and the maximum SWH reached 11 m near the center of Durian. We observed that the typhoon-generated waves propagate across the SCS to farther than 1000 km but only in the forward, the left-frontward, and the right-rearward directions of the typhoon center. The wave energy did not propagate to the left-rearward direction of the typhoon center. This feature implies that the SCS, a highly semi-enclosed marginal sea, can be considered as an independent wave system from nearby oceans except the energy transform through the Luzon Strait.

The last two snapshots in the lower panels are the wave fields after the typhoon departure. It is found that the typhoon-generated waves dispersed, and the monsoon wave characteristics appeared again. To better understand the wave field characteristics, it is necessary to look back at the blended wind fields in Fig. 3. The core of the maximum SWH occurs within the radius of maximum wind. The wave heights are inversely proportional to the distance from the centre of typhoon. And it is found that along the typhoon’s translation track, the core of the maximum SWH is asymmetric with higher SWH and wider core on the right side than the left. Besides, during TY Durian’ passage in the middle of SCS, its moving velocity (3.5 –6.8 m/s) was below but close to the group speed of dominant waves (5.8 –7.6 m/s), so resonant effects exist to some degree. As TY Durian departed on December 5 (Fig. 3k and l), high SWH occurred again west of Luzon (Fig. 4k and l). This is the monsoon swell generated by the large-scale background forcing of NE monsoon winds.

**Figure 5:**
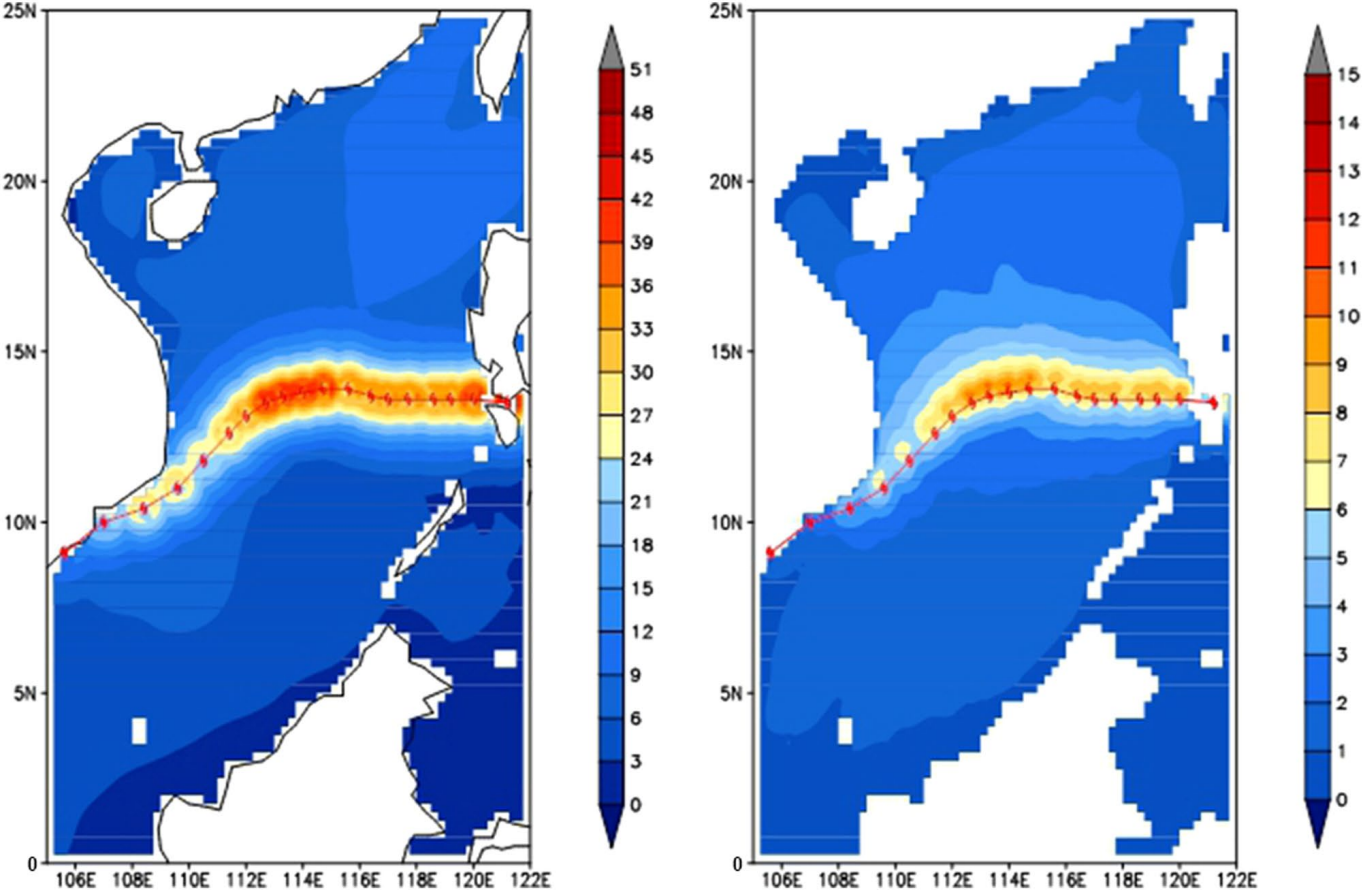
Horizontal distributions during the passage of Typhoon Durian: Left: maximum wind speeds; Right: maximum SWH calculated using NWW3.

Surface waves and the forcing wind relationships are closely interrelated and should be examined together. During TY Durian passing by the SCS from December 1 to 5, process maxima of the forcing wind speed and the NWW3 model calculated SWH are extracted at each grid point and shown in Fig. 5. The maximum wind speeds on the right side of the track are slightly bigger than the left. Whereas, the maximum SWH present more significant difference. The core of the maximum SWH is seriously asymmetric in width and in magnitude along the track of TY Durian with higher SWH on the right side of the track than the left side.

### Directional wave spectra

**Figure 6:**
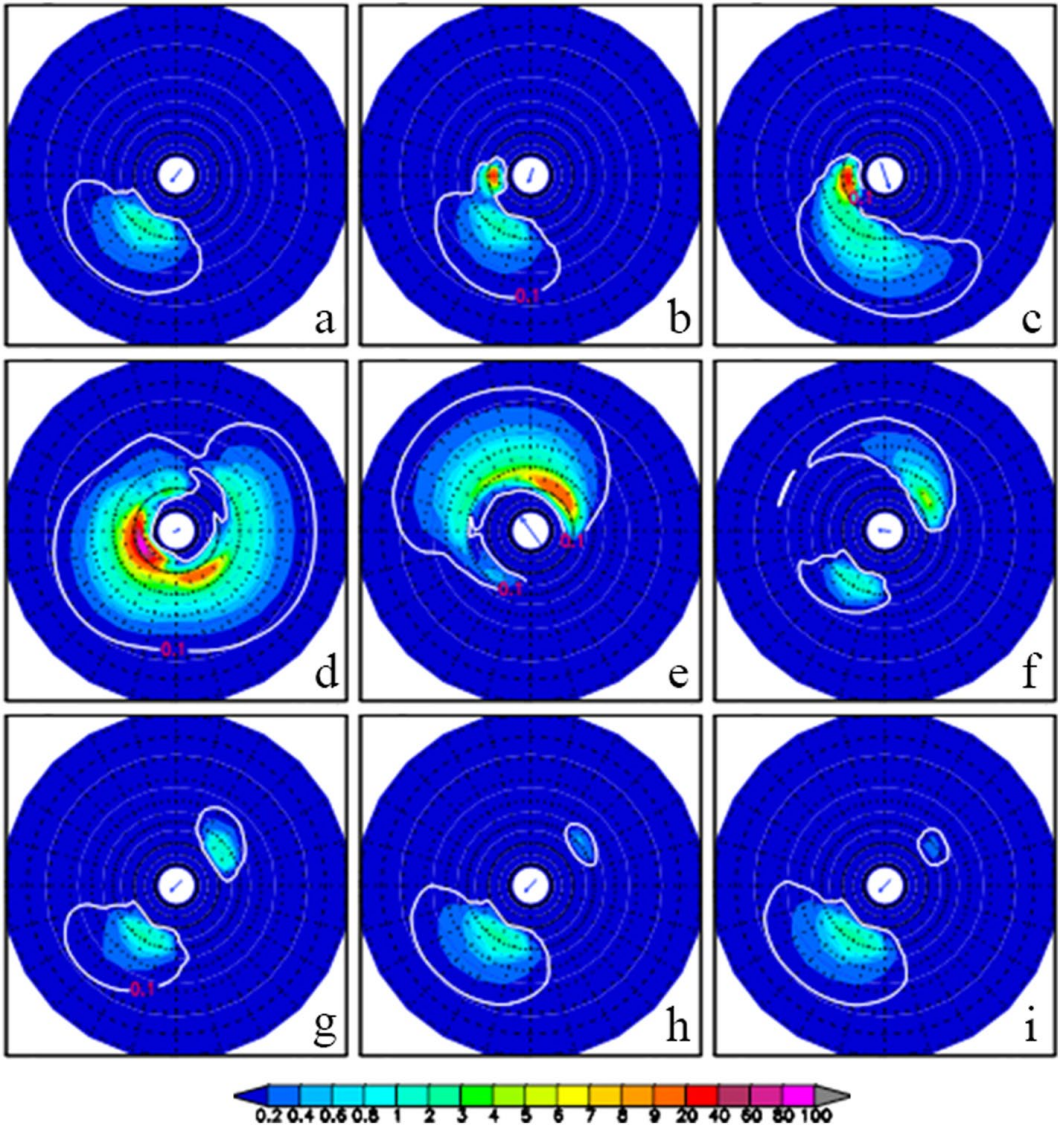
Numerical experiment with NE monsoon winds: 12-h evolution of directional wave spectra at the reference point (114.0° E, 13.8° N) from 1200UTC December 1 to 1200UTC December 5, 2006.

The TY Durian center was located at (114.0° E, 13.8° N) at 0000UTC December 3 when it reached the strongest intensity in the SCS. The lowest center pressure was 960 hPa, the maximum wind speed 40.0 m/s, and RMW 31.17 km. The typhoon moving speed slowed down to 3.53 m/s (see Table 1). We take the location of the typhoon center at 0000UTC on December 3 as the reference point (114.0° E, 13.8° N, and see Fig. 2). Figure 6 shows the twelve-hour evolution of directional wave spectra at the reference point during TY Durian’s passage from 1200UTC December 1 to 0000UTC December 5. The arrow indicates the wind vector at each moment. Before 0000UTC December 2 (Fig. 6a−b), winds blow from the northeast showing the dominant winter monsoon, and waves are mostly generated downwind (southwestward direction). From 0000 to 1200UTC on December 3 when the typhoon center passed by (Fig. 6d−e), waves occur in almost all directions with generation of higher frequency waves. After the departure of TY Durian (Fig. 6g−i), two evident wave packets remain in the southwestward and northeastward directions. However, the typhoon-generated waves (in the opposite direction to monsoon winds) decay rapidly (although they still exist a few days after the typhoon’s departure).

### The effect of monsoon winds: sensitivity experiments without the NE monsoon winds

**Figure 7:**
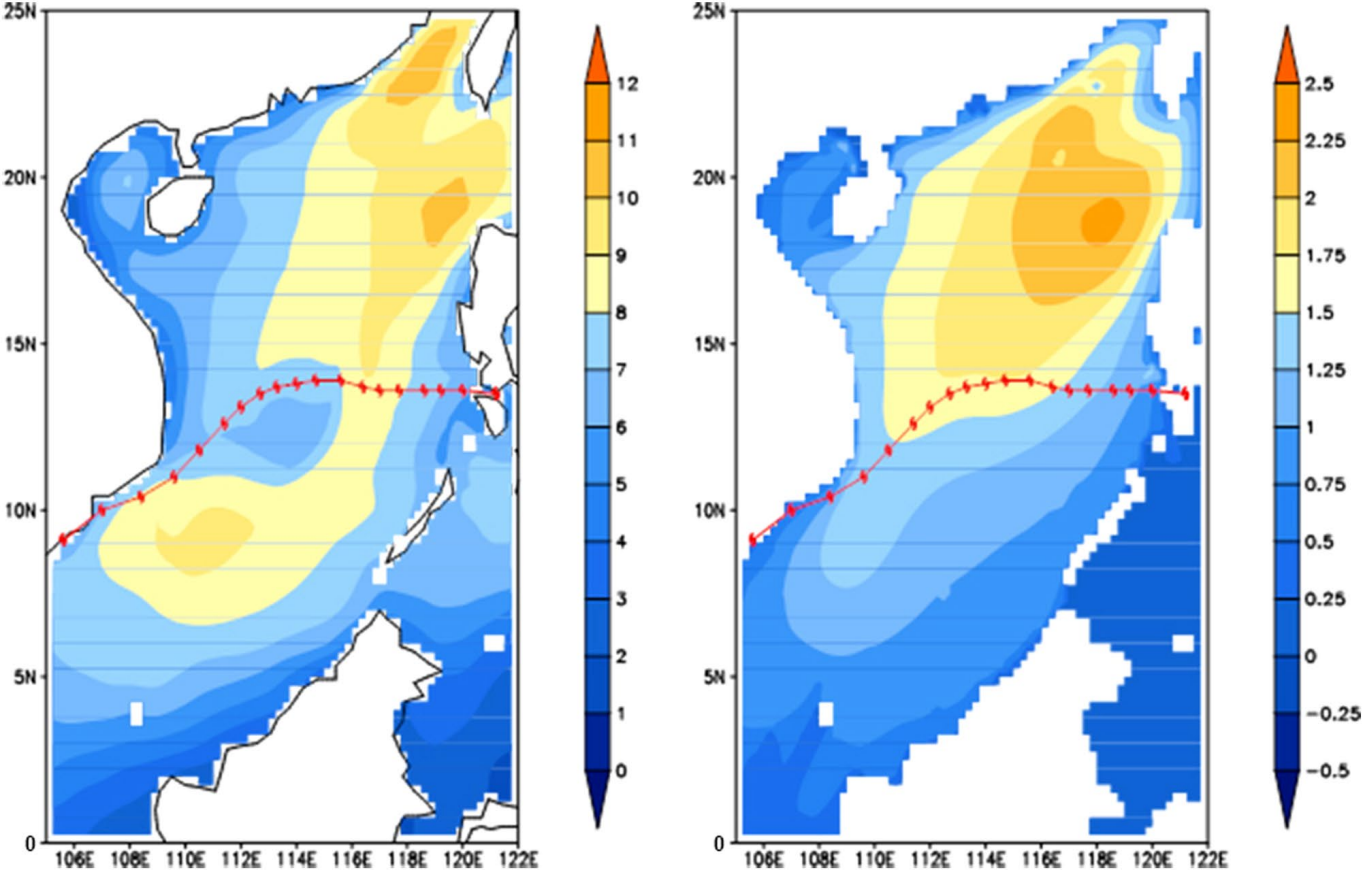
Differences (with NE monsoon winds minus without NE monsoon winds): left: maximum wind speed; right: maximum SWH.

The sensitivity experiment without the NE monsoon winds [i.e., ***V***_bg_ = 0 in Eq. (4)] was carried out to reveal the role of the large-scale NE monsoon winds on the typhoon wave field characteristics in the SCS. The integration time is still from 0000UTC November 25 to 0000UTC December 6. We compare the SCS wave characteristics between with NE monsoon winds and without NE monsoon winds. We use the process maxima of wind speed and SWH at each grid point in the computational domain. The spatial distributions of the difference of with NE monsoon winds minus without NE monsoon winds are shown in Fig. 7. The role of the NE monsoon winds is thus highlighted. Due to topographic influence and the “narrow pipe effect”, wind is strong in the northeast of the SCS, south to Taiwan Strait and west of Luzon Strait. And the monsoon wind effect is distributed mainly in the eastern deep area of the northern SCS (Fig. 7), where larger waves are generated (Fig. 4a−b, and k−l). Meanwhile, we note that the differences are not large near the typhoon track.

**Figure 8:**
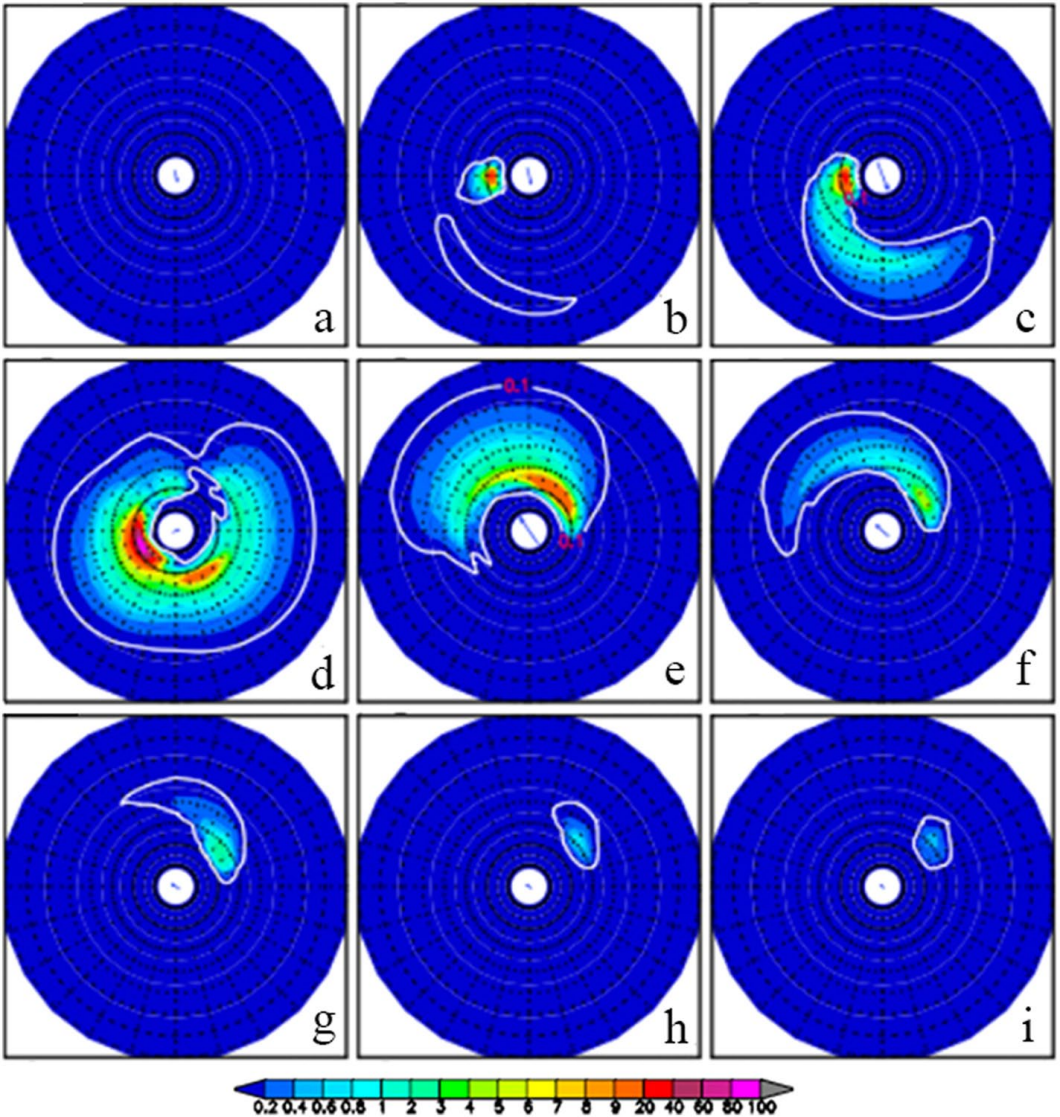
Sensitivity experiment without NE monsoon winds: twelve-hour evolution of directional wave spectra at the reference point (114.0° E, 13.8° N) from 1200UTC December 1 to 1200UTC December 5, 2006.

Figure 8 shows the twelve-hour evolution of directional wave spectra at the reference point by the sensitivity experiment without NE monsoon winds during TY Durian’s passage from 1200UTC December 1 to 0000UTC December 5. Comparisons between the above-mentioned two experiments show the effect of the NE monsoon winds on the directional wave spectra in the SCS. Comparing Figs. 8 to 6, it is found that the two directional wave spectra are almost the same from 1200UTC December 2 to 1200UTC December 3 (c −e on Figs. 6 and 8), but quite different for other time periods. Before being affected by TY Durian at the reference point (e.g., 0000UTC on December 2), there is a high wave energy center in the southwestward direction with the NE monsoon winds (Fig. 6a) but none without the NE monsoon winds (Fig. 8a). One day after the typhoon’s departure from the reference point (e.g., 0000UTC on December 4), the typhoon-generated waves dispersed, and the previous swell energy generated by the monsoon winds appeared once again. This suggests that the monsoon-generated swells do not decay and remain throughout the typhoon period.

## Conclusions

The unique feature of monsoon, typhoon, and monsoon again wave fields in the SCS was studied by wave modeling experiments under the combined wind forcing of prevailing winter monsoon and TY Durian (2006) when it passed westward over the middle of the SCS in early December 2006. The importance of this work lies in that the SCS is both a highly semi-enclosed marginal sea and part of the world-renowned east Asian monsoon climate zone. Here over the SCS, not only winter monsoon stronger and fast moving southeasterly in conjunction with cold air outbreaks (cold surge) , but also late autumn and winter typhoons passing the SCS (mainly in the middle) more frequently. For this purpose, we carried out wave modeling experiments with NE monsoon winds and the sensitivity experiment without NE monsoon winds. The parametric model wind field for the westward migrating typhoon were blended into the large-scale background wind field with the weighted average algorithm for the sake of smoothing. The effects of typhoon and monsoon on the wave characteristics were analyzed. The typhoon effect was simulated using the ideal typhoon wind. The monsoon effect was simulated using the difference of with monsoon winds minus without monsoon winds.

Here we observe that the typhoon-generated waves propagate across the SCS to farther than 1000 km but only in the forward, the left-frontward, and the right-rearward directions of the typhoon center. The wave energy did not propagate to the left-rearward direction of the typhoon center. This feature implies that the SCS, a highly semi-enclosed marginal sea, can be considered as an independent wave system from nearby oceans except the energy transform through the Luzon Strait. We also show that the core of the maximum significant wave height (SWH) is seriously significantly asymmetric in width and in magnitude along the track of TY Durian with higher SWH and wider core on the right side of the track than the left side.

By the difference of with monsoon winds minus without monsoon winds, we found that the NE monsoon winds weakly affect the SWH field near the typhoon track and strongly away from the track, especially in the deep water area of the northern SCS where the NE monsoon winds produce high waves. Our results also demonstrate that before the typhoon arrival, a high wave energy center is found in the southwestward direction with the NE monsoon winds but none without the NE monsoon winds; and after the typhoon departure, the typhoon-generated waves dispersed, and the previous swell energy generated by the steady NE monsoon winds appeared once again. This suggests that the monsoon-generated swells do not decay and remain throughout the typhoon period. Both the typhoon waves and the monsoon-generated swells overlap and add up according to the properties of wave signals. The feature of monsoon, typhoon, and monsoon again wave fields in the SCS is unique. Nature has provided us with the unique natural experimental fields in the SCS for the typhoon wave overlapping monsoon swell research.
